# HGF and Direct Mesenchymal Stem Cells Contact Synergize to Inhibit Hepatic Stellate Cells Activation through TLR4/NF-kB Pathway

**DOI:** 10.1371/journal.pone.0043408

**Published:** 2012-08-23

**Authors:** Pei-pei Wang, Dong-ying Xie, Xu-Jing Liang, Liang Peng, Geng-lin Zhang, Yi-nong Ye, Chan Xie, Zhi-liang Gao

**Affiliations:** 1 Department of Infectious Diseases, the Third Affiliated Hospital of Sun Yat-Sen University, Guangzhou, Guangdong Province, China; 2 Department of Infectious Disease, the First Affiliated Hospital, Jinan University, Guangzhou, Guangdong, China; 3 Department of Infectious Diseases, the First Peoples Hospital of Foshan City, Foshan, Guangdong Province, China; 4 Key Laboratory of Tropical Disease Control, Ministry of Education, Sun Yat-sen University, Guangzhou, Guangdong Province, China; University of Cincinnati, United States of America

## Abstract

**Aims:**

Bone marrow-derived mesenchymal stem cells (BMSCs) can reduce liver fibrosis. Apart from the paracrine mechanism by which the antifibrotic effects of BMSCs inhibit activated hepatic stellate cells (HSCs), the effects of direct interplay and juxtacrine signaling between the two cell types are poorly understood. The purpose of this study was to explore the underlying mechanisms by which BMSCs modulate the function of activated HSCs.

**Methods:**

We used BMSCs directly and indirectly co-culture system with HSCs to evaluate the anti-fibrosis effect of BMSCs. Cell proliferation and activation were examined in the presence of BMSCs and HGF. c-met was knockdown in HSCs to evaluate the effect of HGF secreted by BMSCs. The TLR4 and Myeloid differentiation primary response gene 88(MyD88) mRNA levels and the NF-kB pathway activation were determined by real-time PCR and western blotting analyses. The effect of BMSCs on HSCs activation was investigated in vitro in either MyD88 silencing or overexpression in HSCs. Liver fibrosis in rats fed CCl_4_ with and without BMSCs supplementation was compared. Histopathological examinations and serum biochemical tests were compared between the two groups.

**Results:**

BMSCs remarkably inhibited the proliferation and activation of HSCs by interfering with LPS-TLR4 pathway through a cell–cell contact mode that was partially mediated by HGF secretion. The NF-kB pathway is involved in HSCs activation inhibition by BMSCs. MyD88 over expression reduced the BMSC inhibition of NF-kB luciferase activation. BMSCs protected liver fibrosis in vivo.

**Conclusion:**

BMSCs modulate HSCs in vitro via TLR4/MyD88/NF-kB signaling pathway through cell–cell contact and secreting HGF. BMSCs have therapeutic effects on cirrhosis rats. Our results provide new insights into the treatment of hepatic fibrosis with BMSCs.

## Introduction

Liver fibrosis is the excessive deposition of extracellular matrix and scar formation surrounding damaged liver and this can be effectively reversed [1], [2]. Activated hepatic stellate cells are generated in the extracellular matrix (ECM) of the principal cells during the process of liver fibrosis. ECM production is the primary reason for the excessive fibrosis formation, which eventually leads to cirrhosis. Acquired fibrosis may result from the action of a number of pathogenic factors, toxic exposures, chronic viral hepatitis or the presence of non-alcoholic fatty liver disease. These etiological factors may work separately or in combination with each other to produce cumulative effects [3]. A large number of in vivo experimental and clinical studies have shown that endotoxin levels in patients with liver cirrhosis are significantly increased, and LPS (an endotoxin) can directly activate HSC in vivo. TLR4 is the primary LPS receptor, and TLR4 polymorphisms are closely related to liver fibrosis. Thus, the LPS-TLR4 pathway plays an important role in fibrosis [4]. TLR4 signals through adaptor proteins, including MyD88, to activate down-stream effectors that include NF-kB, mitogen-activated protein kinase (MAPK), and phosphatidylinositol 3-kinase (PI3K). Collectively, these pathways regulate the expression of pro-inflammatory cytokines and genes that control cell survival and apoptosis.

BMSCs are pluripotent stem cells with the potential to differentiate into liver cells. Recent studies have also shown that BMSCs play a substantial role in liver fibrosis treatment without allograft rejection. Studies from animal models have shown that BMSCs infusion ameliorates liver fibrosis and reverses fulminant hepatic failure. A number of clinical trials also proved that BMSCs can effectively alleviate end-stage liver disease and improve symptoms and liver function, indicating the effectiveness and safety of BMSCs in clinical implantation [5]–[7]. However, it has also been reported that BMSCs have the potential to promote fibrosis [8], [9]. Therefore, for therapeutic applications, it will be important to understand the potency and possible repair mechanisms of BMSCs to help us understand the nature of hepatic fibrosis.

Since both the LPS-TLR4 pathway and BMSCs are involved in liver fibrosis, we hypothesized that BMSCs may interfere LPS-TLR4 pathway and inhibit NF-kB activation during fibrosis. To test this hypothesis, we examined the expression of TLR4 in HSCs stimulated with different doses of LPS and investigated the regulatory role of BMSCs in MyD88-mediated LPS-stimulated TLR4 expression.

## Materials and Methods

### Ethics Statement

#### Normal liver and bone marrow samples

Normal liver samples were collected from patients undergoing resection of hepatic hemangiomas at the Department of Hepatobiliary Surgery, the third affiliated hospital of Sun Yat-sen University. Bone marrow suspension cells (10 ml) were obtained from healthy donors in the department of infectious diseases, the third affiliated hospital of Sun Yat-Sen university. All samples were obtained with written, informed consent in accordance with the Sun Yat-sen University Ethical Committee requirements.

### Bone Marrow Separation for Primary Human BMSCs Cultures

BMSCs isolation and culture were described in our previously study [10]. Prior to experimental use, the third generation BMSCs were determined to be CD44, CD90 positive and lack expression of CD45 and CD34 by flowcytometry analysis.

### Human HSCs Isolation and Cell Line Culture

HSCs were isolated by a 2-step collagenase perfusion from surgical specimens of two normal human livers as described previously [11]. All tissues were obtained by qualified medical staff, with donor consent and the approval of the Sun Yat-sen University Ethical Committee requirements. Cell viability was determined using trypan blue exclusion staining. Hepatic stellate cell line LX2 was obtained from the Cancer Center of Sun Yat-sen University. Isolated HSCs and LX2 were seeded on uncoated plastic tissue culture dishes and cultured in Dulbecco’s modified Eagle medium (DMEM, Invitrogen, Carlsbad, CA, USA) supplemented with 10% fetal bovine serum (FBS, HyClone, Logan, UT, USA), 2 mM L-glutamine, 100 units/ml penicillin and 100 ug/ml streptomycin, cultured at 37°C in a humidified atmosphere containing 5% CO_2_.

### Physical Contact Co-culture System and Insert Co-culture System

The GFP-labeled LX2 cells were mixed and co-cultured with BMSCs in supplemented DMEM containing 10% FBS at a density of 1∶1 for LX2 and BMSCs. Since there is no barrier between the two populations, effects are produced from both the exchange of soluble factors and from physical contact. As a comparison to the physical contact co-culture system, LX2 cells and BMSCs were also co-cultured in a bicompartmental system by using 6.5 mm Transwell® with 1.0 µm Pore Polycarbonate Membrane Insert, which were purchased from Corning (Corning, NY, USA). In this case, two types of cells shared the culture medium but were not in physical contact. Cells were seeded in DMEM and incubated overnight. Immediately prior to treatment the medium was changed for fresh DMEM. A total of 2×10^5^ LX2/ml and equivalent volumes of BMSCs releasate and pellet fractions were added. Where indicated, cells were treated with 5 or 50 ng/ml of recombinant HGF (R&D Systems).

### In Vivo Transplantation of BMSCs

Fourty SD rats in six weeks were purchased from the Institute of Materia Medica (Chinese Academy of Sciences, Beijing, China). This study was carried out in strict accordance with the recommendations in the Guide for the Care and Use of Laboratory Animals of the National Institutes of Health. The protocol was approved by the Committee on the Ethics of Animal Experiments of Sun Yat-Sen University. To induce liver cirrhosis, 6.0 ml/kg body weight of carbon tetrachloride (CCl4) mixed with olive oil (1∶1 ratio) was injected intraperitonealy into rats twice a week up to 7 weeks. Five rats were random sacrificed to evaluate if the model was successful. Then the rest rats were divided into two group: BMSC treatment group (25 rats) and control group (10 rats): 1×10^6^ BMSC in 100 ul PBS or 100 ul PBS as a control were injected into tail vein of rats. Three rats were sacrificed at each predetermined time interval (12 h, 24 h, 48 h, 72 h, 144 h) after BMSC post-transplantation to track the BMSC location. CCl4(3 ml/kg) was injected for another two weeks after cell transplantation to maintain persistent liver damage and all the left rats were sacrificed after 3 weeks BMSC post-transplantation. Liver tissue was collected after perfusing with 4% paraformaldehyde solution and preserved in formalin buffer solution for histopathological studies. For protein and total RNA isolation, liver tissue was snap-frozen in liquid nitrogen and then stored at −80°C.

### Hematoxylin and Eosin (HE) and Masson Staining

The rat livers were fixed in formalin for 48 h, paraffin embedded and sectioned into 4-µm thick slices for HE staining or Masson staining. After sealing the slides containing the tissue slices with neutral gum, the stained tissue slices were microscopically examined at 200 × magnification. Subsequently, color images in five randomly chosen microscopic fields of each slice were captured and analyzed by a medical image software program (Image-Pro Plus, Media Cybernetics, USA) to semi-quantitatively determine the areal density (AD) i.e. collagen fiber area over liver area.

### Scheuer Scoring System

A semiquantitative histological score was applied based on the histological classification and quantitation of the Scheuer scoring system using a scale of 0–3 for the inflammatory activity (0, absent; 0.5, minimal; 1, mild; 2, moderate; 3, severe), and 0–4 for the evaluation of fibrosis (0, absent; 1, mild; 2, moderate without septa; 3, moderate with septa; 4, cirrhosis) [12].

### Serum Assay

Blood was taken from the abdominal aorta, and the following indicators were analyzed: total protein (TP), albumin (ALB), alanine aminotransferase (ALT), and aspartate aminotransferase (AST).

Other experimental procedures are available in Materials and Methods S1.

### Statistical Analysis

Data are reported as mean values ± SD. The statistical significance of differences between mean values was determined by Student’s t test. A *P* value of less than 0.05 was considered significant. Statistical calculations were performed using SPSSv16 software (SPSS Inc., Chicago, Illinois, USA).

## Results

### Bone Marrow-derived Cells Expressed Characteristic Surface Markers of BMSCs

**Figure 1:**
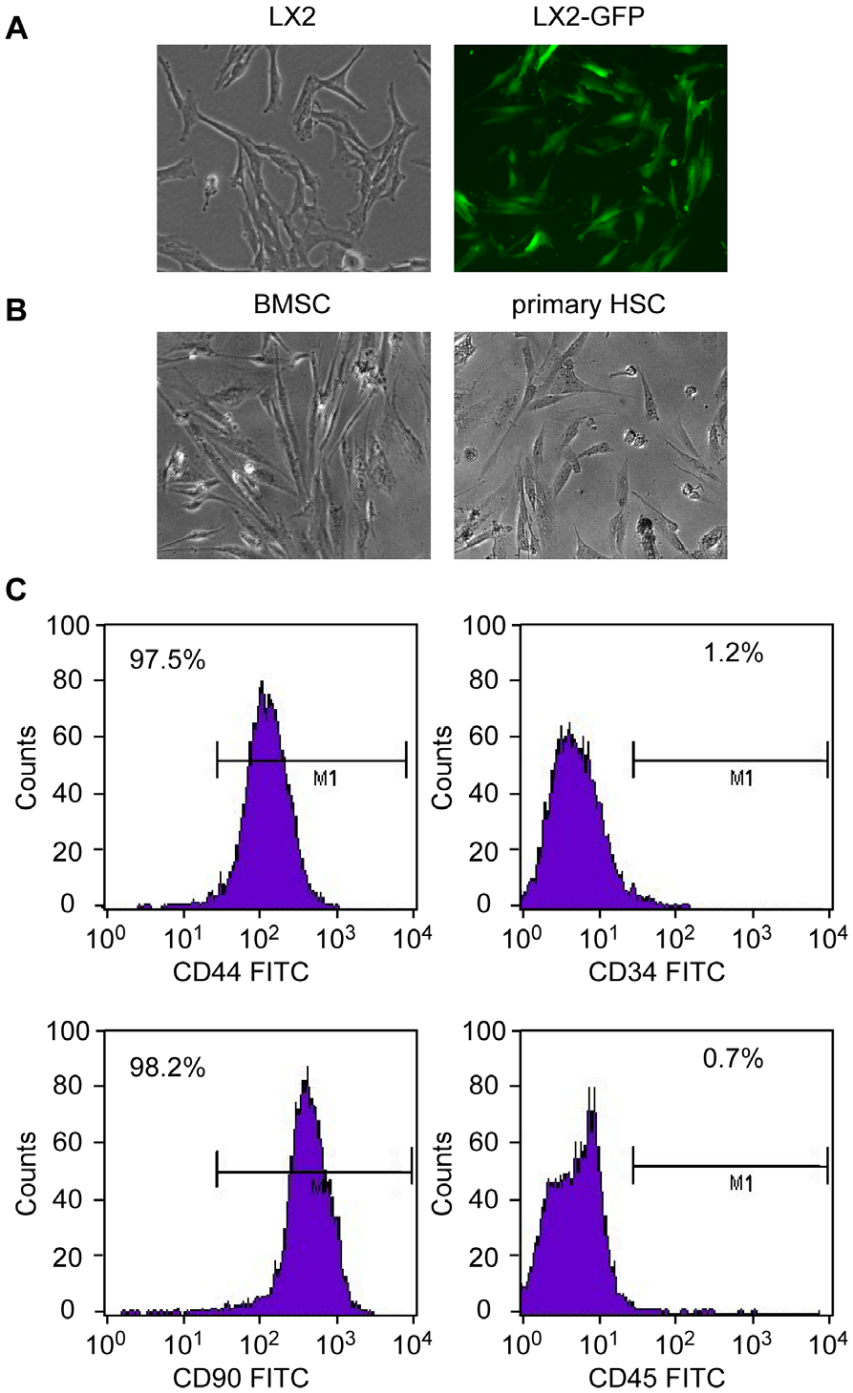
Primary Cells morphology and molecular expression. (A) HSC cell line LX2 (left) and GFP labeled LX2 (right). (B) BMSCs were separated from bone marrow and purified by removing the non-adherent cells at least three times. The 3rd generation BMSCs (medium) were fusiform and fibroblast-like (100×). Primary human HSC (right). (C) FCM analysis showed that the 3rd generation BMSCs expressed CD44 and CD90 but not CD45 or CD34.

BMSCs obtained by bone marrow aspiration and expanded in vitro appeared similar to fibroblasts, with a characteristic spindle-shaped fusiform morphology (Figure 1A). LX2 and primary HSCs were also fibroblasts alike (Figure 1A and 1B). In cultured cells of the 3rd generation analyzed by ﬂow cytometry, more than 97% of the cells expressed CD44 (97.2±1.35%, Fig. 1B) and CD90 (98.0±1.20%, Figure 1C), a surface marker characteristic of BMSCs. The absence of contaminating hematopoietic cells in the BMSCs population was verified by the lack of surface antigens defining hematopoietic progenitor cells (CD34, 1.1±0.03%) or leukocytes (CD45, 0.68±0.05%). Thus, the bone marrow cells after the 3^rd^ passage were of high purity, and expressed CD44^+^, CD90^+^, CD34^−^ and CD45^−^, which are markers for BMSCs.

### LPS induced Activation of LX2 Cells and Upregulation of TLR4

**Figure 2:**
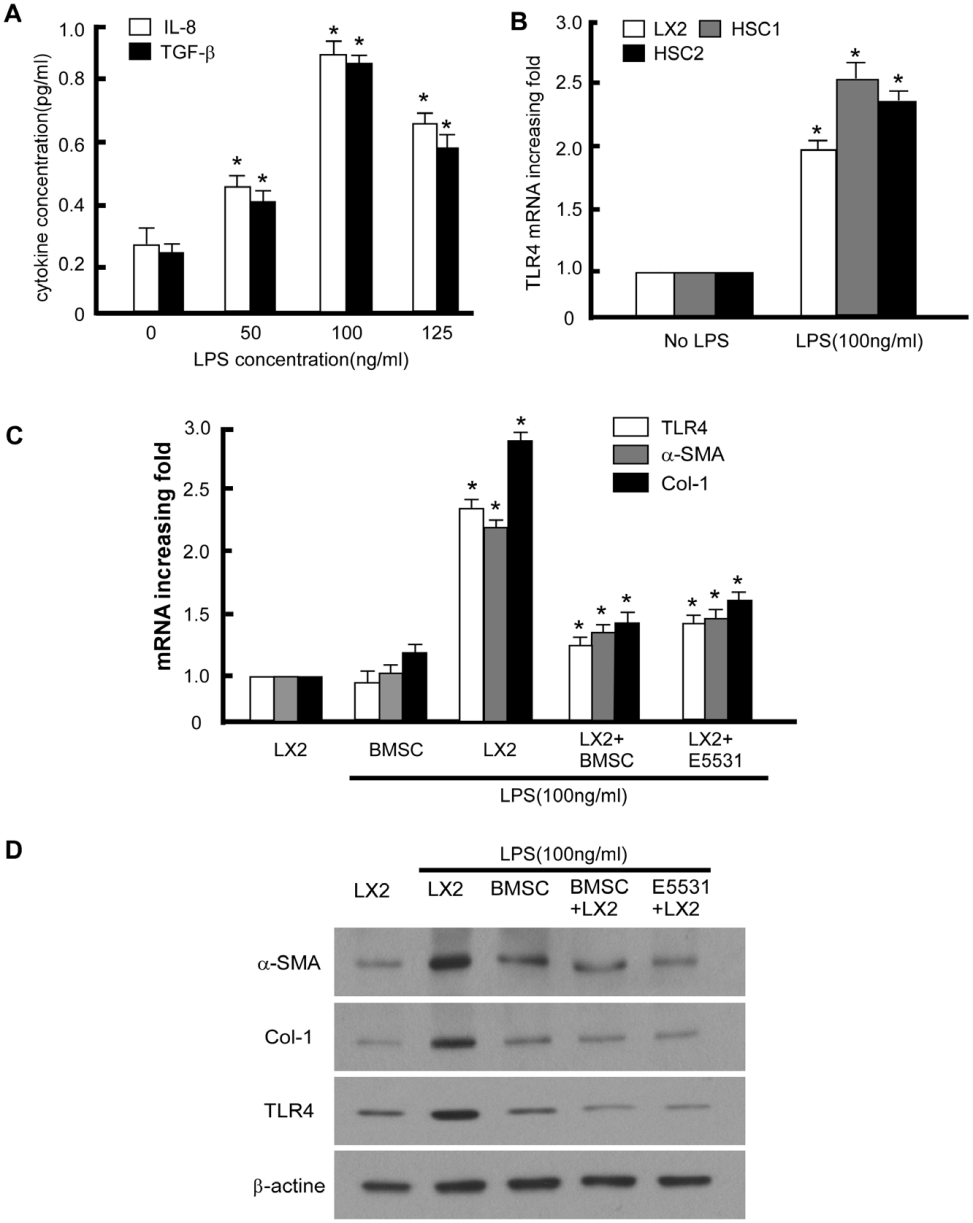
BMSC inhibited LPS induced activation of LX2 and upregulation of TLR4. (A) The effect of different dose of LPS on LX2 cells activation as the IL-8 and TGF-β secretion. All data are shown as mean ± SD from three independent experiments. * P<0.05. (B) Relative mRNA expression of TLR4 in LX2 cells and two HSCs in response to LPS. Data are depicted relative to expression in cells without LPS, which are assigned a value of 1. (C) RT-PCR analysis of the HSC activation marker α-SMA, Col-1 and TLR4 in the indicated treatments. (D) Western blot analysis of the HSC activation marker α-SMA, Col-1 and TLR4 in the indicated treatments. Expression levels were normalized with GAPDH.

We first evaluated the effect of LPS stimulate hepatic stellate cells in vitro. LX2 cells were cultured with different concentrations of LPS (0, 50, 100, 125 ng/ml) to evaluate if there is a dose depended effect. IL-8 and TGF-β which was secreted by activated hepatic stellate cells were measured by ELISA. Results showed quiescent LX2 cells only secreted small amounts of IL-8 (0.33±0.04 pg/ml) and TGF-β (0.31±0.05 pg/ml) (Figure 2A). However, the secretion of IL-8 and TGF-β of LX2 was significantly increased when LPS added. Results of ELISA showed that IL-8 and TGF-β secretion from LPS-activated LX2 cells was 0.88±0.03 pg/ml and 0.74±0.05 pg/ml respectively after 100 ng/ml LPS stimulation (Figure 2A). Furthermore, the expression IL-8 and TGF-β was increased in parallel to increasing concentrations of LPS. When the LPS concentration reached 100 ng/ml, IL-8 and TGF-β expression levels reached up to three times that of the untreated cells. However, as the LPS concentration was increased to 125 ng/ml, a significant amount of cell died. As such, 100 ng/ml LPS were used in all subsequent experiments. Then the expression of TLR4 in two human primary HSCs cell lines and LX2 cells were assessed by RT-PCR. In quiescent LX2 cells and primary HSCs cells (no LPS stimulation), only low levels of TLR4 mRNA were detected. However, TLR4 was substantially up-regulated in LPS-activated primary HSCs cells and LX2 cells for more than 2 times in vitro (Figure 2B). These results suggested that LPS can stimulate HSC activation and TLR4 gene expression.

### BMSCs Inhibit the Activation of LX2 Cells by Interfering with LPS-TLR4 Pathway

Since BMSCs had benefit to liver fibrosis patients, we test if BMSCs also had the effect to inhibit the activation of LX2 in vitro. We co-cultured LX2 with BMSCs separated by transwell to test if BMSC had the same effect on LPS induced LX2 activation. RT-PCR analysis indicated that LX2 and BMSCs without LPS stimulation only expressed small amount of TLR4, a-SMA, and Col-1. However, after LPS stimulation, the levels of TLR4, a-SMA, and Col-1 expression from LX2 cells were significantly increased for more than two times (Figure 2C), while addition of BMSCs significantly reduced the effect. Furthermore, the results of western blot analysis indicated that LX2 cells without LPS stimulation express only small amounts of a-SMA, Col-1, and TLR4, and addition of LPS stimulated increased expression of these proteins, which are known markers of HSCs activation. However, when BMSCs or the TLR4 inhibitor was added, the expression of these proteins from LX2 cells was decreased significantly (Figure 2D). These data suggest that protection of LPS induced LX2 activation by BMSCs was associated with TLR4.

### BMSC Promote Activation of the HGF Pathway in LX2 Activation

**Figure 3:**
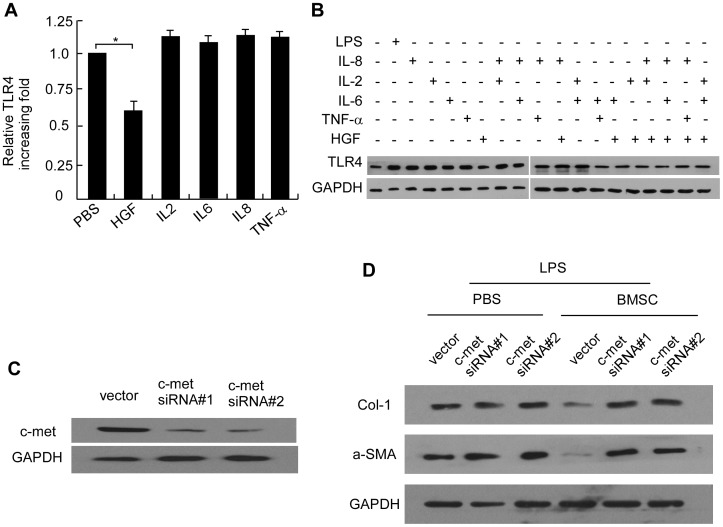
HGF is the major cytokine involved in BMSC-mediated inhibition of LX2 activation. (A) Relative mRNA expression of Col-1 and α-SMA in LX2 with different cytokine stimulation. Data are depicted relative to expression in LX2 cells cultured with PBS, which are assigned a value of 1. All data are shown as mean ± SD from three independent experiments. * P<0.05. (B) Western blot analysis for TLR4 expression under different combination of cytokine. GAPDH was used as a loading control for all Western blots (C) Knockdown of c-met in two specific shRNA-transduced LX2 cell lines. (D) Western blot analysis for Col-1 and α-SMA expression in LX2 with c-met knockdown.

To gain better insight into the signaling pathways involved in BMSCs-to-LX2 cell communication, we detected the effect of BMSCs-secreted cytokines on protection of LPS induced LX2 activation. Of the many cytokines, we tested HGF, IL-8, IL-2, IL-6, and TNF-a for their ability to effect Col-1 and a-SMA expression of LX2. The RT-PCR showed only HGF decreased Col-1 and a-SMA mRNA expression in LX2 cells for more than two times (Figure S1A). Considering that a prolonged exposure to HGF protects fibrosis in LX2, we investigated whether BMSC-derived HGF could inhibit fibrosis in LX2 cells. As shown in Figure S1B-1E, the mRNA and protein level of Col-1 and a-SMA in LPS stimulated LX2 cells decreased by approximately 1.5- to 2-fold within 6 to 24 hours after HGF treatment and increasing dose of HGF from 20 to 50 ng/ml, demonstrating that effect of HGF on LX2 cells. We next investigated whether HGF protection of LX2 cell is dependent on TLR4 signaling. Interestingly, When LX2 cells were treated with different cytokines for 6 h, we found only HGF induced decrease in TLR4 mRNA expression (Figure 3A). We also combined HGF with other cytokines to evaluate the co-effect on LX2. Western blot showed that quiescent LX2 cells (without LPS stimulation) only expressed a small amount of TLR4. While after LPS stimulation, TLR4 expression increased obviously. Then we used IL-8, HGF, IL-2, IL-6, and TNF-a stimulated LX2 along, and found only HGF deduced TLR4 expression. Besides of these, we combined two or three cytokines together and stimulate LX2, and the results of western blot showed that only when the combination included HGF, there was an effect of TLR4 down-regulation. The combination without HGF demonstrated no effect. Furthermore, no synergistic effect was found when HGF was used together with other cytokines (Figure 3B).

Then we knockdown the HGF receptor, c-met, of LX2 cell and investigate if LX2 activation inhibited by BMSCs was through HGF. As Figure 3C indicated, two c-met siRNA knockdown c-met protein in LX2 cell. When using BMSCs treating LX2/c-met- RNAi, we found that LPS induced LX2 activation could not be suppressed (Figure 3D). The results conferred BMSCs protected LX2 cells from LPS through secreating HGF.

### HGF and Direct BMSC-LX2 Cell Contact Synergize to Inhibit LX2 Activation Gene Expression

**Figure 4:**
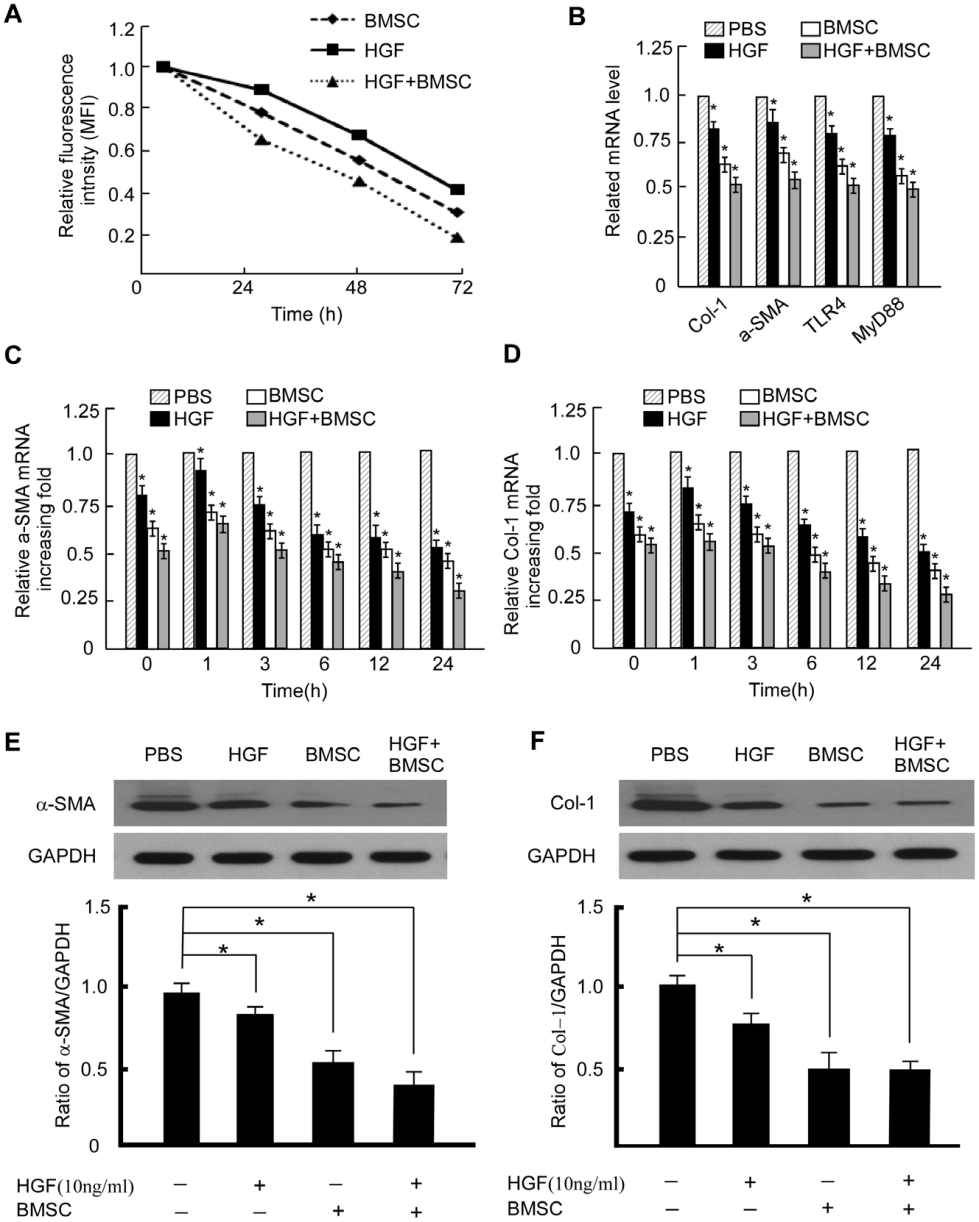
HGF and direct BMSC-LX2 cell contact synergize to inhibit LX2 activation gene expression. (A) MFI of GFP at the indicated times during HSC culture. (B) Relative mRNA expression of Col-1, a-SMA, MyD88 and TLR4 in LX2 with the indicated treatments. Data are depicted relative to expression in LX2 cells with PBS stimulation, which are assigned a value of 1. All data are shown as mean ± SD from three independent experiments. * P<0.05. (C) Real-time PCR show that expression of α-SMA and Col-1(D) in LX2 cells with indicated treatments in different time point. Expression levels were normalized with GAPDH. Western blot analysis of a-SMA (E) and Col-1(F) in LX2 cells with the indicated treatments.

It is believed that effective treatments aimed at suppressing the activation and proliferation of LX2 would reduce the deleterious effects of LX2 on the progression of fibrosis. The proliferation of GFP labeled cells leads to a progressive reduction in mean fluorescence intensity (MFI). We therefore calculated the in vitro growth rate of LX2 by MFI after interaction with BMSCs. Flow cytometry analyses suggested that the MFI of GFP gradually decreased with incubation time during LX2 growth and that direct co-cultured LX2 exhibited relatively slow proliferation rates compared to monocultured cells (Figure 4A).

When GFP-labeled LX2 cells and BMSCs were co-cultured with direct cell-cell contact, the fluorescence labeled LX2 cells was inherited by daughter cells after cell division, but was not transferred to adjacent BMSCs in the co-culture population. Fluorescence-activated cell sorting was then performed to separate fluorescence-positive cells (LX2) and fluorescence-negative cells (BMSCs). Co-cultured cells (3×10^6^) were sorted and 0.9−1.2×10^6^ of each type was obtained after cell sorting. The relative mRNA expression levels of a-SMA, Col-1, TLR4, MyD88 in LX2 co-culture with physical contact, and in the insert co-culture system (no physical contact) versus LX2 alone with HGF are significant deduced compared to PBS control group (Figure 4B). In the LX2 co-culture with physical contact with BMSCs group, the gene relative expression levels were much lower than the groups which were in the insert co-culture system and LX2 alone with HGF. Results for time and dose-depend effect of the three kinds of treatments on LX2 cells using RT-PCR are also shown (Figure 4C and 4D). Genes that were down-regulated at least one half are identified in the BMSCs physical contact co-culture and HGF. The patterns of gene expression for cells exposed to contact co-culture are different from co-culture that only permitted soluble interactions only (i.e. the insert co-culture system). WB also showed the same Synergize effect of HGF and direct BMSC-LX2 cell contact (Figure 4E and 4F). Taken together, these results show that BMSC-derived HGF is necessary to deduce the expression of several fibro-activation genes in vitro but that, in addition, BMSCs provide other signals through physical contacting which synergize with HGF signaling.

### The NF-kB Signaling Pathway is Inhibited by BMSCs in a Contact-dependent Manner and Cooperates with HGF Signaling to Inhibit LX2 Activation

**Figure 5:**
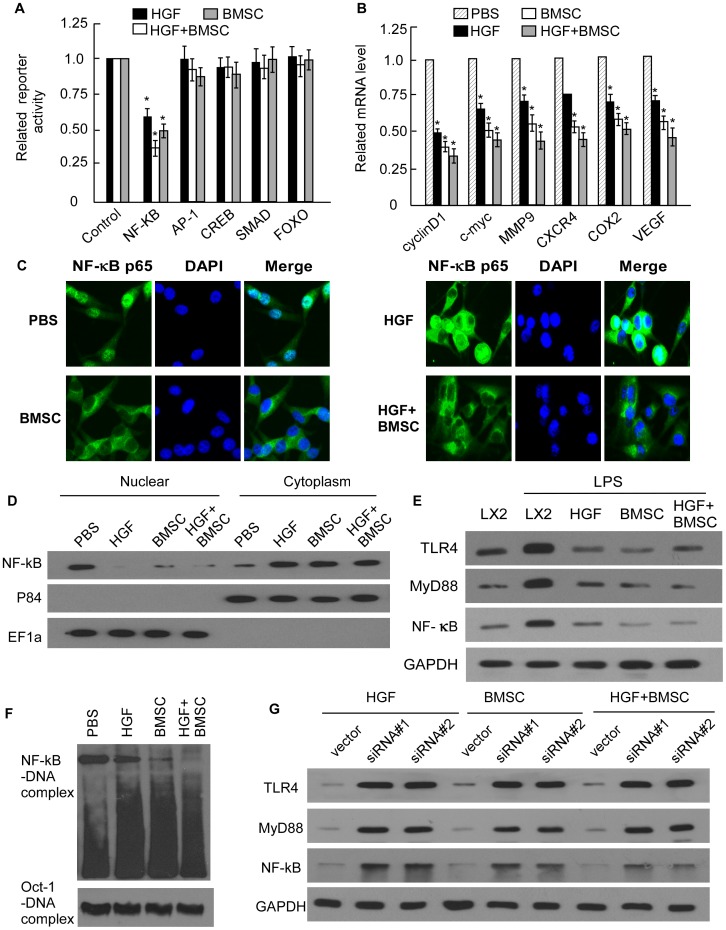
NF-kB signaling pathway is activated by BMSC in a contact-dependent manner and cooperates with HGF signaling to inhibit LX2 activation. (A) Relative luciferase activity in the LPS stimulated LX2 cell with BMSC in a Contact-Dependent Manner (BMSC), BMSC in a contact-dependent manner with HGF addition (BMSC+HGF) and HGF stimulation alone (HGF). (B) Real-time PCR show the expression of cyclinD1, c-Myc, Mmp9, CXCR4, Cox2 and VEGF in LX2 cells with indicated treatments. Expression levels were normalized with GAPDH. (C) Doubling staining with anti-p65 antibody and DAPI was performed, and the NF-kB localization was identified in LX2 cells with indicated treatments (400×). (D) Cytoplasmic and nuclear levels of NF-kB in LX2 cells with indicated treatments were analyzed by WB. (E) WB analysis of TLR4, MyD88 and NF-kB in LX2 cells with the indicated treatments. (F) NF-κB DNA binding activity was analyzed by EMSA Shown is a representative blot from three independent experiments. (G) MWB analysis of TLR4, MyD88 and NF-kB in LX2 cells with c-met knockdown.

We next sought to define the molecular pathway induced upon BMSCs contact that cooperate with HGF to inhibit fibro-activation gene expression. Using a set of luciferase reporter assays for several pathways, we screened for their activation in LPS treated LX2 cells in response to interaction with BMSCs or HGF (Figure 5A). Interestingly, co-incubation with BMSCs or HGF addition decreased activation of the NF-kB pathways in LX2 cells (Figure 5A). Activation of the NF-kB pathway was decreased when cells were incubated with either BMSCs or the pellet fraction from BMSCs. RT-PCR further proved the NF-kB downstream genes including cyclinD1, c-Myc, Mmp9, CXCR4, Cox2 and VEGF, were inhibited by BMSCs, HGF or HGF+BMSC (Figure 5B). NF-kB is composed of the p50 and p65 subunits, and in resting cells the NF-kB complex is sequestered in the cytosol by an inhibitory subunit, IkB. Once stimulated, IkB is phosphorylated and degraded, which allows NF-kB to translocate to the nucleus and induce the expression of its target genes. So we did p65 staining to evaluate the effect of BMSCs on NF-kB signaling. As indicated in Figure 5C, LPS-treated LX2 cells demonstrated primarily nuclear NF-kB staining, whereas exposure to BMSC or HGF for 1 h resulted in a rapid translocation of NF-kB to the cytoplasm. The effect was enforced by co-cultured with BMSCs directly with HGF addition. Additionally, when we examined the cellular localization of NF-kB expression within the cell, we noted a decrease in nuclear NF-kB expression in BMSC or HGF treated cells, and the effect was also enforced by co-cultured with BMSCs directly with HGF addition (Figure 5D). WB further proved the protection of BMSCs or HGF in LPS induced LX2 activation through inhibited TLR4/M yD88/NF-kB signaling (Figure 5E). To determine if BMSC had the protection ability in LPS induced NF-κB DNA binding in LX2, we performed EMSA analysis on nuclear lysates from LX2. As showed in Figure 5F, nuclear NF-κB constitutively bound DNA in LPS-stimulated LX2. While BMSCs and HGF repressed DNA binding of NF-kB. The effect was also enforced by co-cultured with BMSCs directly with HGF addition. When we knockdown c-met in LX2 cells, the protection ability of BMSCs, HGF or director BMSCs co-culture on LX2 cell were significantly impaired (Figure 5G). These data proved the ability of BMSCs to prime LX2 cells activation depends on the synergistic interaction between the NF-kB and HGF signaling pathways, which is triggered by direct BMSC-tumor cell contact.

### BMSC Inhibited the Activation of LX2 Cells Depended on MyD88

**Figure 6:**
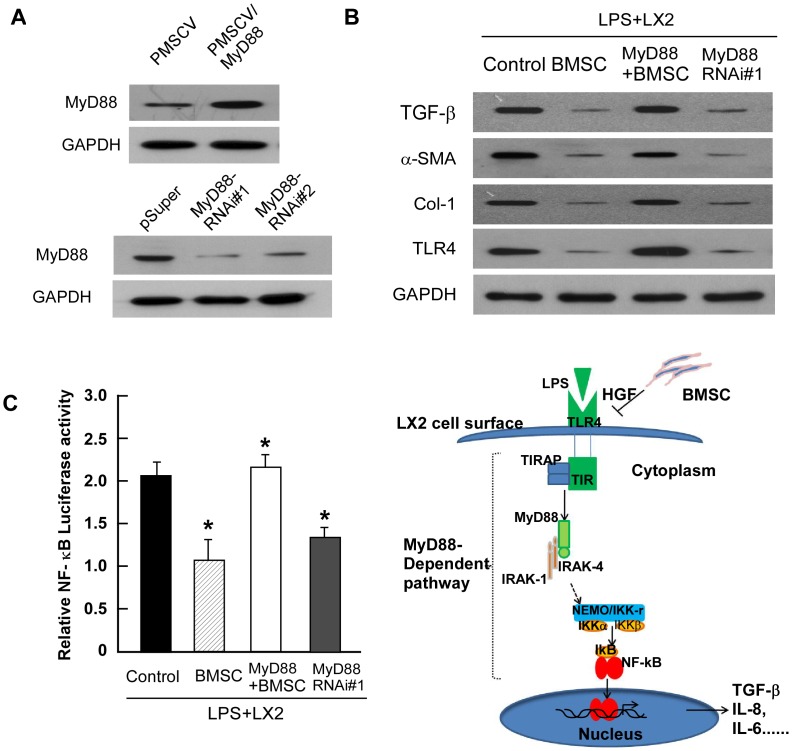
MyD88 was necessary for BMSCs to protect LPS induced activation of LX2. (A) Western blotting analysis of MyD88 expression in indicated cells. GAPDH was used as a loading control. (B) TGF-β, a-SMA, Col-1 and TLR4 was analysis by western blotting on LPS stimulated LX2 cell under BMSC treatment, MyD88 overexpress (MyD88) and MyD88 RNAi#1. (C) Relative NF-kB luciferase activity in the LPS stimulated LX2 cell with BMSC treated and MyD88 overexpress and MyD88 shRNA. (D) Proposed scheme for BMSC inhibition in LPS-stimulated signaling in the activation of LX2.

TLR4 acts as a receptor for LPS and mediates its intracellular actions via the adapter molecule MyD88. Experiments in MyD88 knockout mice demonstrated that MyD88 is required for induction of liver injury in response to hypoxia and LPS [14], [15]. So we further examining the levels of MyD88 and NF-kB in LPS induced activated LX2 cells response to BMSCs. The MyD88 and NF-kB was highly expressed in LPS induced activated LX2 cells based on RT-PCR (Figure 4B). While when BMSCs or HGF was added, the expression of MyD88 and NF-kB of LX2 was significantly decreased. Since MyD88 was important in LPS-TLR4 pathway and responded to BMSCs treating, we further investigate if MyD88 is essential for BMSC protecting LPS induced activation of LX2. We established MyD88-overexpressing or -silenced LX2 stable cell line (Figure 6A). Inhibition of LPS-induced LX2 activation by BMSCs was significantly decreased when MyD88 was overexpressed (LX2/MyD88 cells) (Figure 6B). When MyD88 was knockdown in LX2 cells, the LPS stimulated activation of LX2 was inhibited as treated by BMSCs alone (Figure 6B). The luciferase activity of NF-kB in activated LX2 was also blocked by BMSCs or when MyD88 knockdown, while overexpressing MyD88 rescued the inhibited effect, suggesting that these markers of stellate cell activation were directly regulated by MyD88-dependent TLR4 signaling. Interestingly, the LPS induced luciferase activity of LX2 was inhibited when MyD88 was down-regulated in LX2, suggesting the involvement of TLR4 in this process (Figure 6C and 6D).

### In Vivo Determination of BMSC-protected HSC Activation and Liver Fibrosis

**Figure 7:**
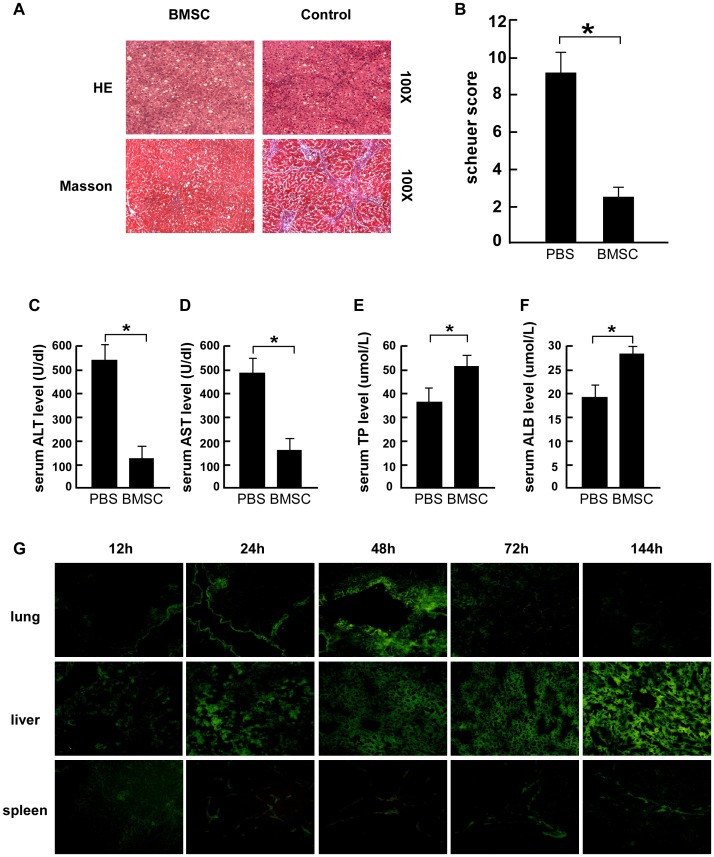
BMSCs transplantation reduced CCl4-induced hepatic fibrosis. (A) Hematoxylin and eosin (up panel) and Micrographs of Masson’s trichrome staining (down panel) of liver paraffin sections from control group and BMSCs group (100X). (B) Scheuer score was quantified. Each value is the mean±SEM of determinations in 10 rats of each group. *P<0.05 compared with PBS control group. Rats serum ALT (C), AST (D), TP (E) and ALB (F) level in each group. (G) Tracking GFP-labeled BMSCs in lung, liver and spleen frozen sections (12 to 144 h following transplantation) using fluorescence microscopy (100×).

After 8 weeks of CCl_4_ injection, liver fibrosis was observed (Figure 7A) and proved by HE and Masson-stained sections. The Scheuer score in control group was 9±1.25 compared 3±0.37 in BMSCs transplantation group (*P*<0.05) (Figure 7B). So BMSCs transplantation had 2-fold decrease of fibrotic area and ameliorated the formation of liver fibrosis (Figure 7B). Rats with fibrosis treated with PBS alone had 4-fold increase of serum ALT (533.6±45.1 U/dl) (Figure 7C) and 2.5-fold increase of serum AST (489.7±42.8 U/dl) (Figure 7D) activity relative to those with BMSCs group (ALT 136.1±38.6 U/dl, *P*<0.05 and AST 152.3±10.3 U/dl, *P<*0.01), respectively. The TP of the rats treated with PBS alone was 38.2±7.1 umol/L, and 50.1±7.8 umol/L for the rats treated with BMSCs (Figure 7E). ALB level of the rats treated with BMSCs was 28.7±3.4 umol/L compared to18.5±3.8 umol/L in PBS treated rats (Figure 7F). The results suggested that BMSCs transplantation ameliorates the CCl4-induced deterioration of liver function. Meanwhile, GFAP- labeled BMSCs were strongly and diffusely present near the expanding septa and in the perisinusoidal spaces of sidual hepatic parenchyma when frozen tissue sections from these livers since 24 h after BMSCs injection and proliferated at 144 h were observed under fluorescence microscopy (Figure 7G); however, no fluorescent cells were observed in sections from the untreated cirrhotic rats and few BMSCs were stayed at lung and spleen. These results suggest that BMSC labeled with GFP in vitro are able to survive in the livers of cirrhotic rats without effect other organs. Thus, BMSCs transplantation relived liver fibrosis safe and effect.

## Discussion

In this study, we found that BMSCs inhibited LX2 cell activation in vitro and relieved liver fibrosis in vivo. Furthermore, we showed that the anti-fibrotic effect of BMSCs was through induction of HGF and cell-to-cell contact. C-met knockdown or MyD88 over expression reversed the BMSCs inhibition of NF-kB luciferase activation. These data suggested that BMSCs plays an important role in protecting LPS induced LX2 activation via inhibition of the LPS-TLR4-MyD88-NF-kB pathway and may represent a novel therapeutic way for the disease.

After liver injury, LPS levels increase in the portal and systemic circulation, owing to changes in the intestinal mucosal permeability and increased bacterial translocation. Animal studies have shown that LPS can increase hepatic fibrosis [13], [14]. Patients with liver cirrhosis of Child-Pugh A class, B class, and C class, followed by LPS increased [15]. TLR4 is an important member of the TLR family and a receptor for LPS, has been found in the portal vein of chronic hepatitis patients and expressed in HSCs and KC cells too [16]. Although both LPS and TLR4 has an important role in liver cirrhosis, the mechanism is still unclear. A recent report documents the discovery of a single nucleotide polymorphism (T399I) in TLR4 that can significantly reduce the risk of hepatic fibrosis [17]. As such, therapeutic targeting of this pathway may block the formation of fibrosis and has important research value. Our experiments also indicated that LPS activated LX2 cell, which significantly increased TLR4 as well as collagen synthesis. TLR activation can stimulate two main pathways. One is dependent upon the adapter protein MyD88 (MyD88-dependent pathway) and the other is not (MyD88-independent pathway). The differential responses mediated by distinct TLR ligands and had different effect. Our results showed the protection of BMSCs on LX2 was MyD88 depended and found that at least part of the effects of BMSCs may have resulted from the suppression of NF-kB activation, which would normally occur in response to MyD88-dependent ligand-induced stimulation of TLRs. In the present study, the MyD88-overexpressing LX2 cells demonstrated increased NF-kB activity even when co-cultured with BMSCs and MyD88-knocdown decreased NF-kB activity of LX2 even without BMSCs protection. These findings confirmed the critical role of TLR4-MyD88 signaling in regulation of LX2 cell activation and identified novel biologic pathways affecting the risk of hepatic fibrosis progression. In our experiments, LX2 cell activation was inhibited when these cells were co-cultured with BMSCs, and BMSCs inhibit LX2 activation through inhibiting the LPS-TLR4-MyD88 pathway.

Animal experiments have shown that BMSCs can effectively relieve fibrosis, improve liver function, and effect the remodeling of fibrous tissue, which may be related to the integration of bone marrow cells and the expression of matrix metalloproteinase 9 [18]–[22]. In addition, transplantation of BMSCs can result in a large increase in the number of liver and bile duct cells, subsequently improving liver function. We have previously reported good results with autologous transplantation of BMSCs through the hepatic artery for treatment of patients with liver failure [23]. To evaluate the function of BMSCs on HSCs, we further conclude from in vivo experiments that BMSCs confer their liver protective effects. This suggests that BMSCs can evoke endogenous repair mechanisms in liver. Indeed, BMSCs are capable of producing a variety of cytokines and hematopoietic growth factors [24]–[26]. Among the great number of growth factors liver protective effects have been most attributed to HGF. HGF is a potent growth factor involved in liver regeneration that has various effects on epithelial and nonepithelial cells. HGF is a polypeptide originally characterized as a highly potent hepatocyte mitogen [27], [28]. HGF also suppresses the onset of severe hepatic injury and maintains the integrity of hepatocytes in the livers of mice with cholestasis induced by alphanaphthylisothiocyanate [29]–[31]. Using a transwell co-culture assay, our study suggested that BMSCs protected activation of HSCs. This effect was mediated in part through paracrine signaling by HGF secreted by BMSCs. When we knockdown HGF receptor c-met, the protection of BMSCs and HGF decreased. Our results could therefore indicate that HGF production is upregulated in BMSCs-CM compared to DMEM, inhibiting NF-kB pathway which is important in LPS induced LX2 activation.

Importantly, labeled LX2 and BMSCs directly co-culture inhibited LPS induced LX2 activation much more effect. These results present the possibility of direct interactions between activated HSCs and BMSCs through direct cell-to-cell contact, although we and others have previously investigated paracrine regulation between these two cells. In this study, we therefore propose a new potential mechanism of the anti-fibrotic effect of BMSCs. The present data indicate that BMSCs in direct cell-cell contact not only result in growth inhibition in HSCs compared with the transwell system, but also suppress HSCs activation in vitro. This observation provides evidence that cell–cell adhesion is involved in the HSCs growth inhibition by BMSCs. In particular, directly co-cultured BMSCs displayed stronger inhibitory activities compared with the paracrine blockade effect in two-chamber co-culture models.

In summary, BMSCs are able to protect against liver fibrosis induced by LPS. Directly co-culture, in contrast to the indirectly co-culture of BMSCs, provides the most effective treatment to prevent LX2 activation. These results are due to the fact that BMSCs can secret HGF, which, in turn, can modulate the host immune response and homeostasis between TLR4 and NF-kB pathway. The results of this study provide several new insights into reversing hepatic fibrosis through the anti-fibrotic effects of treated BMSCs targeting HSCs in vitro. The direct co-culture system we have established was applied to explore the effect of direct interplay between BMSCs and HSCs involving paracrine signaling as well as cell–cell adhesion and juxtacrine signaling. Regardless of the mechanism, our study is the first to demonstrate that induction of the TLR4-MyD88-NF-kB signaling pathway can dramatically repress proliferation of HSCs co-cultured with BMSCs in vitro. But the in vivo effect of BMSCs inhibited HSC activation through TLR4-MyD88-NF-kB pathway is still unclear. Therefore, more studies needed to clarify the mechanism and make BMSCs more effectively to reduce liver inflammation and reduce scar formation, thus inhibiting liver fibrosis and improving the quality of life of patients and prolonging their survival time.

## Supporting Information

Figure S1 (A) Relative mRNA expression of TLR4 in LX2 with different cytokine stimulation. Data are depicted relative to expression in LX2 cells cultured with PBS, which are assigned a value of 1. (B) Real-time PCR show that HGF induces Col-1 and a-SMA mRNA expression in a dosage-dependent manner. (C) Real-time PCR show that expression of Col-1 and α-SMA in LX2 cells with HGF (50 ng/ml) stimulation in different time point. Expression levels were normalized with GAPDH. (D, E) Western blot analysis for a dosage and time -dependent (HGF 50 ng/ml) effects of HGF on Col-1 and a-SMA expression.
(TIF)

Materials and Methods S1 (DOCX)
