# Supplementary figures and images for: HGF and Direct Mesenchymal Stem Cells Contact Synergize to Inhibit Hepatic Stellate Cells Activation through TLR4/NF-kB Pathway
Source: PLoS One. 2012 Aug 23;7(8):e43408. doi: 10.1371/journal.pone.0043408 (PMC3426540; doi:10.1371/journal.pone.0043408)

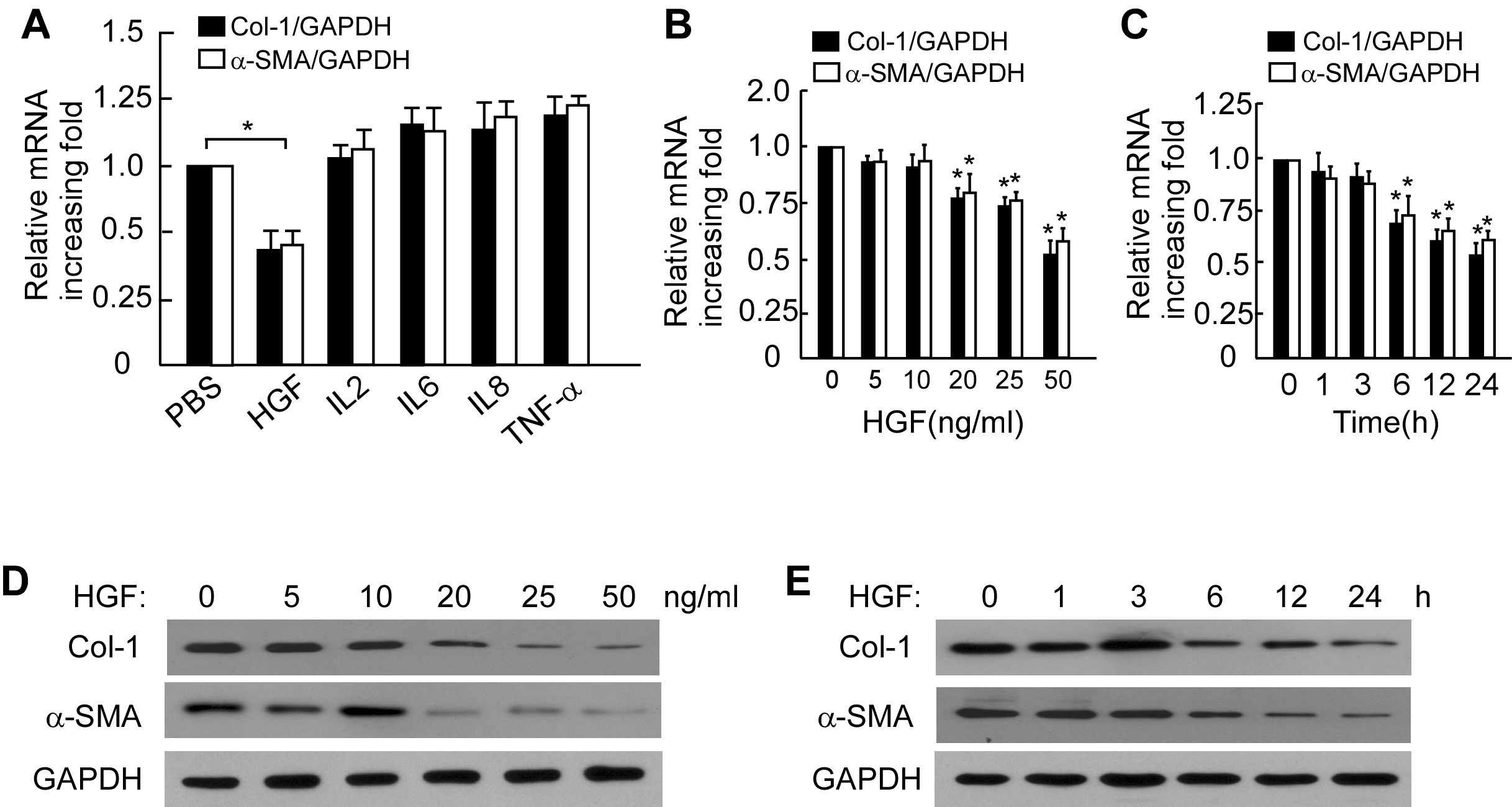

Supplement: Figure S1 — (A) Relative mRNA expression of TLR4 in LX2 with different cytokine stimulation. Data are depicted relative to expression in LX2 cells cultured with PBS, which are assigned a value of 1. (B) Real-time PCR show that HGF induces Col-1 and a-SMA mRNA expression in a dosage-dependent manner. (C) Real-time PCR show that expression of Col-1 and α-SMA in LX2 cells with HGF (50 ng/ml) stimulation in different time point. Expression levels were normalized with GAPDH. (D, E) Western blot analysis for a dosage and time -dependent (HGF 50 ng/ml) effects of HGF on Col-1 and a-SMA expression. (TIF) [file pone.0043408.s001.tif]
