# Supplementary material for: HGF and Direct Mesenchymal Stem Cells Contact Synergize to Inhibit Hepatic Stellate Cells Activation through TLR4/NF-kB Pathway
Source: PLoS One. 2012 Aug 23;7(8):e43408. doi: 10.1371/journal.pone.0043408 (PMC3426540; doi:10.1371/journal.pone.0043408)
Supplement: Materials and Methods S1 — (DOCX) [file pone.0043408.s002.docx]

**Supplemental material and methods**

**Flow cytometry analysis and fluorescence-activated cell sorting.**

Flow cytometric analysis of co-cultured cells was performed on an Epics XL-MCL cytometer (Beckman Coulter, Fullerton, CA). The fluorescence was detected by using excitation at 488 nm and diachronic/band long pass filters. The results were analyzed with instrument software System II 3.0. Fluorescence-activated cell sorting was performed by using an Elite ESP cell sorter (Beckman Coulter), which is fitted with four lasers and provides emission wavelengths ranging from 350 to 647 nm. The droplet cell-sorting function was used to separate fluorescence-positive cells from fluorescence-negative cells.

**Cell proliferation analysis**

Labeled-LX2 was co-cultured with BMSCs for 3 days and was separated by fluorescence-activated cell sorting (FACS). The cell growth was measured by relative mean fluorescence intensity (MFI). Reports have indicated that there is an inverse relationship between GFP-labeled cell division and MFI and that extensive cell proliferation results in a progressive, step-wise reduction in MFI. LX2 monoculture was used as a control. Each treatment was performed at least three times.

**Assessment of LPS responsiveness in LX2 cells**

LX2 cells were seeded into the upper wells of a 24-well Boyden chamber (Transwell; pore size, 1.0 μm; Corning, Inc., Corning, NY) at 2×10^5^ cells per well, cultured at 37ºC with 5% CO_2_. The experiment was divided into five groups: BMSCs + LX2 cells (with 100 ng/ml LPS), LX2 cells (with 100 ng/ml LPS), BMSCs (with 100 ng/ml LPS), LX2 cells (no LPS), and LX2 cells (with 100 ng/ml LPS and 10 uM TLR4 inhibitor E5531 (EISAI Co., Tokyo, Japan). When the LX2 cells were 90% confluent, the culture medium was changed to serum-free medium for 12 h. A total of 100 ng/ml of LPS and/or BMSCs (2×10^5^) was added to the appropriate bottom wells. The LX2 cells in the indicated group were pretreated with 10 uM E5531 for 30 min at 37°C before addition of DMEM medium containing 100 ng/ml LPS. Samples from the different cultures system were analyzed in triplicate for each condition. The cells were then treated with 100 ng/mL LPS in 0.2% bovine serum albumin (BSA) or 0.2% BSA alone for 12 h prior to isolation of messenger RNA (mRNA) and protein analysis.

**Plasmids**

To label LX2 and BMSC with GFP fluorescence, LX2 and BMSC cells were stable infected with pMSCV-GFP-puro plasmid. For overexpression of MyD88, human MyD88 sequences were cloned into the pMSCV-retro-puro plasmid to generate pMSCV-retro-MyD88, the sequences were retroviral production and infection was performed as standard protocols. For depletion of MyD88, human siRNA sequence was cloned into the pSuper-retro-puro plasmid (Promega, Madison, WI, USA) to generate pSuper-retro-MyD88-RNAi, the sequence was RNAi#1: AACAGACAGACTATCGGCTTA and RNAi#2: AACAGAAGCGACTGATCCCTA (synthesized by Invitrogen). Transfection of small interfering RNA (siRNA) into cells was conducted when the cells reached 70% confluence. The siRNAs of MET and a non-targeting control were purchased from Santa Cruz Biotechnology. Experiments were conducted according to the manufacturers' instructions. The reporter plasmid for quantitatively detecting the transcriptional activity of NF-κB, FOXO, AP-1, CREB, SMAD were generated in the pGL3-Enhancer plasmid (Promega, Madison, WI, USA) as described previously [13].

**Enzyme-linked immunosorbent assay (ELISA)**

Supernatants were collected after the BMSC were cultured for 48 h and stored at -20ºC. ELISAs for IL-8, HGF, IL-2, IL-6, TGF-β and TNF-α were performed according to the manufacturer’s protocol (International Immuno-Diagnostics, Foster City, CA, USA). All samples from the different time points (from three separate cultures) were analyzed in triplicate for each condition.

**Electromobility Shift Assay (EMSA)**

Nuclear lysates from a confluent monolayer of LX2 were extracted using the nuclear extract kit (Active Motif) following the manufacturer’s instructions. DNA protein interactions were then investigated using the Lightshift chemiluminescent EMSA kit (Pierce). Briefly, LX2 cells were lysed in hypotonic lysis buffer (20 mM Hepes, pH 7.5; 5 mM NaF, 10 µM Na2MoO4, 0.5% NP-40 and 0.1 mM EDTA), and then nuclei were resuspended in lysis buffer supplemented with 0.5 mM DTT and 0.2 mM PMSF. The NF-κB consensus oligonucleotides (sense: AGTTGAGGGGACTTTCCCAGGC; antisense:GCCTGGGAAAGTCCCCTCAACT) labeled with 32P by T4 polynucleokinase (Promega, Madison, WI) were incubated with nuclear extracts in binding buffer (10 mM Tris pH 7.6, 1 mM DTT, 0.5 mM EDTA, 2 µg polydI-dC and 10% Glycerol) at 30°C for 30 minutes. The free DNA and DNA-protein mixtures were resolved in 5% native polyacrylamide gels in 0.5× TBE buffer (45 mM Tris, 45 mM boric acid and 1 mM EDTA, pH 8.3) by electrophoresis. The gel was dried and subjected to autoradiography analysis.
**Western blot test**

Cells from each group were cultured for 48 hours in each group were collected and Western blotting was done using standard protocols. Primary antibodies against human smooth muscle actin (α-SMA, santa cruz, 1:1000), human collagen I antibody (Col-1, abcam, 1:1000), human myeloid differentiation primary response gene 88 (MyD88, R & D, 1:1000), human GAPDH (R & D, 1:5000), human TLR4 (R & D, 1:1000), NF-kB p65 antibody (Rockland, Gilbertsville, PA, 1:1000), TGF-β(R & D, 1:1000) and c-met antibody (R & D, 1:1000). Both of which were conjugated to horseradish peroxidase secondary antibodies, where appropriate. Blots were developed using enhanced chemiluminescence (ECL, Dura, Pierce).

**Real-time polymerase chain reaction (RT-PCR)**
 Cells from each group were cultured for 48 h, collected, and RNA was extracted using Trizol reagent (Invitrogen, Carlsbad, CA, USA) and reverse-transcribed with a reverse transcription system (Promega, Madison, WI, USA) according to the manufacturers’ protocols. DNA was extracted from the cell pellets using an extraction kit (Qiagen, Hilden, Germany). An initial amplification using specific primers was performed with a denaturation step at 95ºC for 10 min, followed by 35 cycles at 95ºC for 60 s, 58ºC for 30 s, and 72ºC for 30 s; this was followed by 72ºC for 5 min and storage at 4ºC. RT-PCR analysis utilized an ABI Prism 7000 Sequence Detection System and the SYBR Green PCR Master Mix (Applied Biosystems, Foster City, CA, USA). The relative expression of each gene was normalized to that of GAPDH. PCR primers for TLR-4,sense 5-GTCCATCGGTAGATCTAG GGA-3, antisense 5-AGAATTGCCAGCCATATATAA-3; IL-8 sense 5-AGAGTGGACCACACTGCGC-3, antisense 5-ACATCCCAACGGTCTACGTTA-3; MyD88 sense 5-CACT CGCAGTTTGTTGGATG-3, antisense 5-CCACCTGTAAAGGCTTCTCG-3; NF-κB sense 5-GGGGACTACGACCTGAATG-3, antisense 5-GGGCACGAT TGTCAAAGAT-3; GAPDH sense 5’-ACCACAGTCCATGCCATCAC-3’ and antisense, 5’-TCCACCA C CCTG TTGCTGTA -3’; cyclinD1 sense 5’- AACTACCTGGACCGCTTCCT-3’, and antisense 5’-CCACTTGAGC TTGTTCACCA-3’;c-myc sense 5’-TTTCGGGTAGTGGAAAACCA-3’, and antisense 5’-CACCGAGTCGTAGTCGAGGT-3’; MMP9 sense 5’-ACGACGTCTTCCAGTACCGA-3’, antisense 5’-TTGGTCCACCTGGTTCAACT-3’; CXCR4 sense 5’-AGGAAGCTGTTGGCTGAAAA-3’, and antisense 5’-CTCACTGACGTTGGCAAAGA-3’; COX2 sense primer 5’-TCTCCAACCTCTCCTACTAC-3’, antisense primer 5’-GCACGTAGTCTTCGATCACT-3’; VEGF sense 5’-CTACCTCCACCATGCCAAGT-3’, and antisense 5’-AGCTGCGCTGATAGACATCC-3’; Col-1 sense 5’- CACACGTCTCGGTCATGGTA -3’, antisense 5’- AAGAGGAAGGCCAAGTCGAG -3’; a-SMA sense 5’- CCAGAGCCATTGTCACACAC -3’, antisense 5’- CAGCCAAGCACTGTCAGG -3’.

**Immunofluorescence**
 Immunoﬂuorescence analyses were performed for NF-kB p65 localization. In brief, the cells were grown on glass cover-slips, and treated for 1h with LPS (100ng/ml). Then BMSCs, HGF or BMSCs plus HGF were added in the well. Cells were then washed, ﬁxed and permeabilized. After incubation with a 1:500 dilution of rabbit anti-human NF-kB p65 antibody (Rel A; Rockland, Gilbertsville, PA) for 1 h at room temperature, the cells were washed three times in phosphate buffered saline-tween (0.1%), followed by incubation with FITC-conjugated goat anti-rabbit IgG (Jackson Immuno Research Laboratories) and 4,6-diamidino-2-phenylindole (DAPI, Sigma) in the dark for 10 minutes. At last, the cells were observed and photographed with a ﬂuorescence microscope (NIKON 80i, Japan).

**Luciferase Assay**

A total of 15,000 LX2 cells/50 ml Opti-MEM were plated in a 96-well Cignal Finder Multi Pathway reporter Array plate as recommended by the manufacturer (SABiosciences) and incubated for 24 hr. The medium was then changed and cells were treated with BMSCs, HGF or BMSCs + HGF. The ratios of Fireﬂy to Renilla Luciferase activities (relative light units [RLU]) were measured in cell lysates with the Dual Luciferase Reporter Assay System (Promega) 24 hr after treatment with BMSCs, HGF or BMSCs + HGF.

**Preparation of Cytoplasmic and Nuclear Fractions**

Cells were scraped with cold PBS and resuspended in HLB buffer (10 mM HEPES [pH 7.9], 10 mM KCl, 1.5 mM MgCl2, 0.5 mM DTT). The cells were then Dounce-homogenized and centrifuged at 1000 × g for 5 min at 4ºC. The supernatant (cytoplasmic fraction) was stored at–80°C until use. The nuclei in the pellet were isolated by centrifugation and resuspended in nuclear extraction buffer (20 mM HEPES [pH 7.9], 420 mM NaCl, 1.2 mM MgCl_2_, 0.2 mM EDTA, 25% glycerol) for 30 min at 4°C. After centrifugation at 15,000 × g for 30 min at 4ºC, the supernatant (nuclear fraction) was stored at –80°C.
